# Platelets protect lung from injury induced by systemic inflammatory response

**DOI:** 10.1038/srep42080

**Published:** 2017-02-03

**Authors:** Shuhua Luo, Yabo Wang, Qi An, Hao Chen, Junfei Zhao, Jie Zhang, Wentong Meng, Lei Du

**Affiliations:** 1 Department of Cardiovascular Surgery, West China Hospital, Sichuan University, Chengdu, Sichuan, 610041 China; 2 Department of Laboratory Medicine, West China Hospital, Sichuan University, Chengdu, Sichuan, 610041 China; 3 Department of Anesthesiology and Translational Neuroscience Center, West China Hospital, Sichuan University, Chengdu, Sichuan, 610041 China; 4 Laboratory of Stem Cell Biology, West China Hospital, Sichuan University, Chengdu, Sichuan, 610041 China

## Abstract

Systemic inflammatory responses can severely injure lungs, prompting efforts to explore how to attenuate such injury. Here we explored whether platelets can help attenuate lung injury in mice resulting from extracorporeal circulation (ECC)-induced systemic inflammatory responses. Mice were subjected to ECC for 30 min, then treated with phosphate-buffered saline, platelets, the GPIIb/IIIa inhibitor Tirofiban, or the combination of platelets and Tirofiban. Blood and lung tissues were harvested 60 min later, and lung injury and inflammatory status were assessed. As expected, ECC caused systemic inflammation and pulmonary dysfunction, and platelet transfusion resulted in significantly milder lung injury and higher lung function. It also led to greater numbers of circulating platelet-leukocyte aggregates and greater platelet accumulation in the lung. Platelet transfusion was associated with higher production of transforming growth factor-β and as well as lower levels of tumour necrosis factor-α and neutrophil elastase in plasma and lung. None of these platelet effects was observed in the presence of Tirofiban. Our results suggest that, at least under certain conditions, platelets can protect lung from injury induced by systemic inflammatory responses.

Systemic inflammatory responses, easily triggered by extracorporeal circulation (ECC), are a frequent, serious problem because the triggering of inflammatory and coagulation cascades, together with endothelial damage, can lead to multiple organ injury^1^. The lungs are among the organs most vulnerable to this type of injury because they contain an extensive capillary bed and abundant immune cells.

Key drivers of systemic inflammatory-induced pulmonary dysfunction are leukocyte activation and release of pro-inflammatory factors such as tumour necrosis factor (TNF)-α. TNF-α acts as a chemotactic signal to recruit circulating leukocytes, which accumulate inappropriately in lung tissue^2^. During these processes, platelets may also become activated, leading adhesive proteins on their surface, which include P-selectin and β3-integrin (also known as aIIbβ3 or GPIIb-IIIa), to bind to leukocytes. The resulting platelet-leukocyte complexes induce platelets to release various alpha-granule proteins, including coagulation factors, chemokines, and mitogenic factors^3^,^4^,^5^, which influence neutrophil activity. For example, activated thrombin interacts with protease-activated receptor 1 on platelets^6^, inducing P-selectin expression on the platelet membrane. This surface P-selectin interacts with glycoprotein PSGL-1 on neutrophils to give rise to platelet-leukocyte aggregates (PLAs). These aggregates alter the distribution of CD11b/CD18 (Mac-1) on neutrophils, promoting their crawling and recruiting them to inflamed vessels^7^ and to inflamed lung tissue^8^. In these processes, platelets exert strong pro-inflammatory effects that damage tissue. Indeed, depleting platelets in animal models of lung injury ameliorates tissue damage^7^.

In contrast to their pro-inflammatory effects, platelets can also exert anti-inflammatory effects. Alpha granules contain the anti-inflammatory factor transforming growth factor (TGF)-β^9^. Platelets release interleukin (IL)-10 that inhibits TNF-α secretion by monocytes^10^. They also inhibit the secretion of inflammatory mediators from macrophages via a mechanism involving cyclooxygenase type (COX) 1/2 during sterile and bacterial systemic inflammation^11^. The *in vivo* conditions under which platelets exert anti-inflammatory effects are unclear, as are the mechanisms involved.

Platelet count falls under certain inflammatory conditions such as during cardiopulmonary bypass^12^, and transfusion of fresh platelets can prevent bleeding after cardiopulmonary bypass. These considerations led us to wonder whether and how platelets might protect against acute pulmonary dysfunction induced by a systemic inflammatory response. We examined this question using our previously characterized mouse model in which ECC leads to systemic inflammatory response, causing pulmonary dysfunction similar to that seen in humans^13^.

## Results

### Platelet transfusion attenuates ECC-induced lung injury

**Figure 1:**
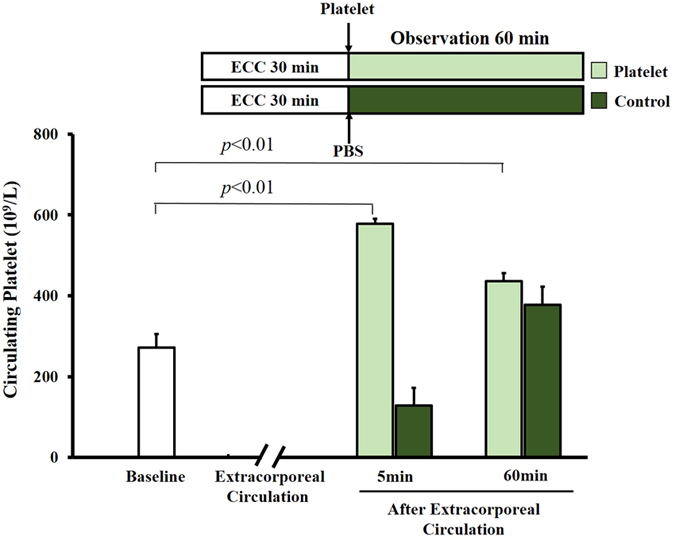
Platelet counts in mice subjected to ECC after fresh platelet transfusion. Mice were subjected to ECC for 30 min, then given PBS (0.1 ml) or platelets (0.1 ml, 3 × 10^8^ from 1.5 ml blood) and sacrificed 5 or 60 min later (n = 20 per group). Platelet counts were determined. As a baseline group, 20 mice were sacrificed after 60 min without ECC. Data shown are mean ± SEM.

Mice were subjected to ECC for 30 min, then treated with phosphate-buffered saline (PBS) or platelets and sacrificed at 60 min after ECC. Animals from both groups were also sacrificed at 5 min after ECC in order to count platelets immediately after ECC. In control animals, platelet count dropped by 50% from before ECC to immediately afterwards (Fig. 1). Platelet count was 3.5-fold higher in the transfused animals than in control animals immediately after ECC, and this difference was significant (p = 0.01) even at 60 min after ECC.

**Figure 2:**
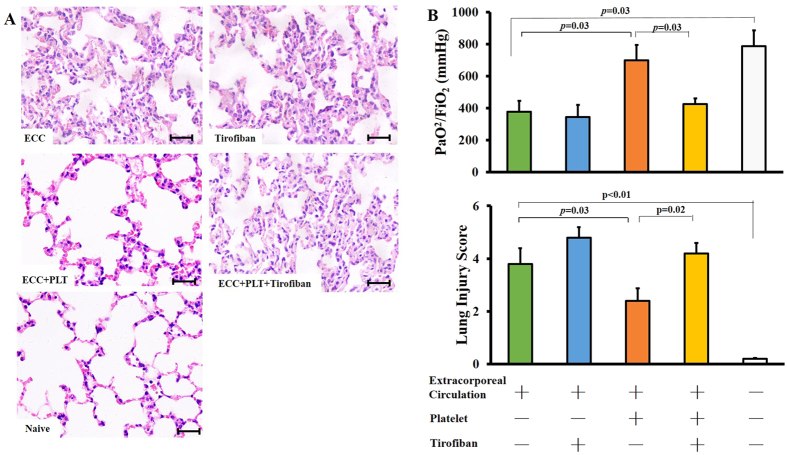
Platelet transfusion attenuates ECC-induced lung injury. Mice were subjected to ECC for 30 min, then treated with PBS, platelets (PLT), Tirofiban, or the combination of platelets and Tirofiban (ECC + PLT + Tirofiban, n = 10 per group). As controls, 10 mice (Naive) did not undergo any intervention. At 60 min after the various treatments, lung tissues were harvested (**A**), and organ function (**B**) and lung injury score (**C**) were evaluated. Lung sections were stained with hematoxylin and eosin and images were captured. Scale bar, 100 μm. Data shown are mean ± SEM.

Consistent with results we reported previously^13^, ECC caused severe lung injury, which was observed as thickening of the alveolar wall, leukocyte infiltration (Fig. 2A), lower PaO_2_/FiO_2_ (Fig. 2B) and higher lung injury score than at baseline (Fig. 2C). This lung injury resembles the lung injury induced by systemic inflammatory responses^14^. Transfusion of fresh unactivated platelets significantly enhanced pulmonary function (p = 0.03) and mitigated lung injury (p = 0.02; Fig. 2A and C). These effects of platelets were eliminated when they were transfused together with Tirofiban, which blocks the GP-IIb/IIIa integrin on the platelet surface.

**Figure 3:**
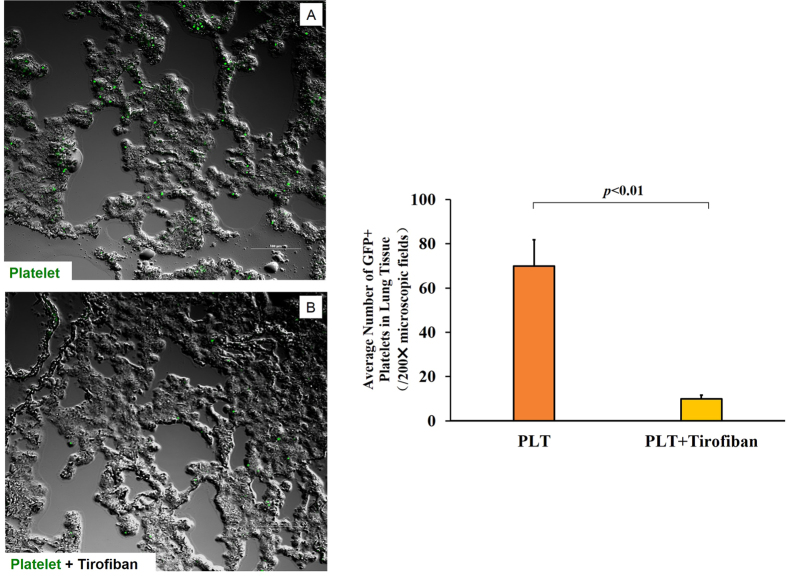
Platelet migration into lung tissue. Mice(n = 4) were subjected to ECC for 30 min, then injected with platelets (3 × 10^8^) from GFP+ mice in the absence (**A**) or presence (**B**) of Tirofiban. At 1 h later, the left lung was harvested and fixed overnight at 4 °C in 4% paraformaldehyde. Paraffin-embedded sections were examined by confocal microscopy. The number of GFP+ platelets in lung tissue was counted in 200 fold microscopic fields. Scale bar, 100 μm. Green dots: GFP+ platelets.

We wanted to examine the possibility that, in our mouse model of lung injury, platelets adhere to activated endothelium via GP-IIb/IIIa^15^ and migrate to lung tissue to help repair it, as shown in other systems^16^. We exposed mice to ECC for 30 min, then injected them with green fluorescent protein-labelled (GFP+) platelets in the presence or absence of Tirofiban. Abundant GFP+ platelets were found in interstitial lung tissue in the absence of Tirofiban (Fig. 3A), while they were rarely observed in its presence (Fig. 3B,C).

### Platelet transfusion attenuates ECC-induced pulmonary inflammation

**Figure 4:**
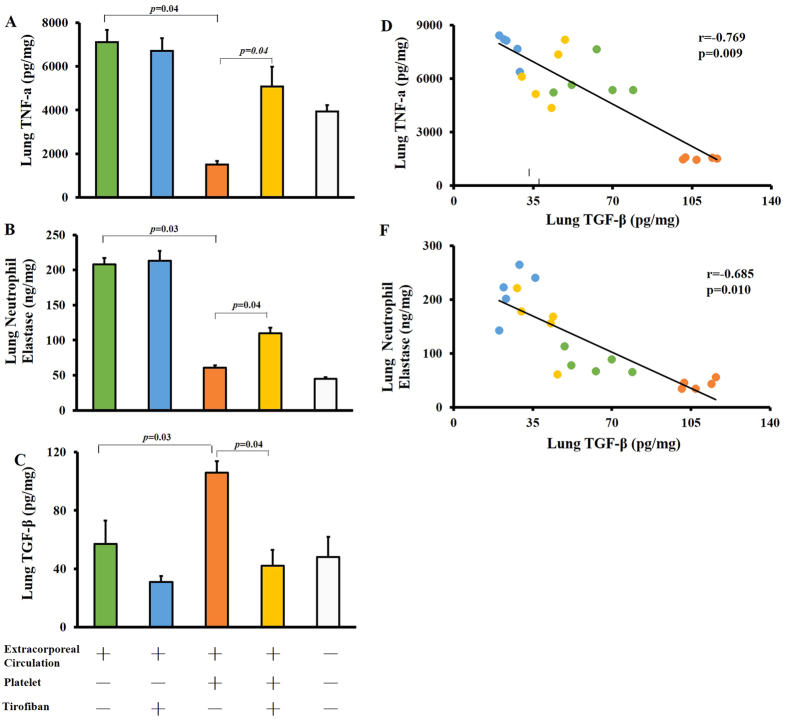
Platelet transfusion attenuates ECC-induced pulmonary inflammation. Mice were subjected to ECC for 30 min, treated as described in the legend to Fig. 2, and then the right lung was harvested 1 h later, homogenized, and centrifuged. Samples were assayed for TNF-α (**A**), neutrophil elastase (**B**), and TGF-β (**C**) using commercial ELISA kits. Levels of TGF-β correlated negatively with TNF-α (**D**) and with neutrophil elastase (**E**). Data shown are mean ± SEM.

Next we wanted to investigate possible mechanisms by which platelet transfusion can alleviate ECC-induced lung inflammation. ECC up-regulated TNF-α in lung tissue, which platelet transfusion significantly reduced (p = 0.04, Fig. 4A). Similarly, ECC also increased levels of neutrophil elastase (NE), which platelet transfusion reduced (p = 0.03, Fig. 4B). These inhibitory effects of platelets were reversed by Tirofiban. These results correlated with the ability of platelet transfusion to reduce leukocyte infiltration in lung interstitial space, and the ability of Tirofiban to increase it. Release of pro-inflammatory factors such as TNF-α from pulmonary monocytes induces leukocyte infiltration in lung tissue^17^.

Platelets produce the anti-inflammatory factor TGF-β^17^, which can regulate a broad range of immune cells^18^. Therefore we assayed the concentrations of TGF-β in lung tissue following treatment with platelets in the presence or absence of Tirofiban. Consistent with their observed anti-inflammatory effects, platelet transfusion led to significantly higher pulmonary TGF-β concentration (p = 0.03, Fig. 4C) and concomitantly lower levels of TNF-α (p = 0.009, Fig. 4D) and NE (p = 0.01, Fig. 4E). These results suggest that TGF-β attenuates ECC-induced lung inflammation.

### Platelet transfusion attenuates ECC-induced systemic inflammation

**Figure 5:**
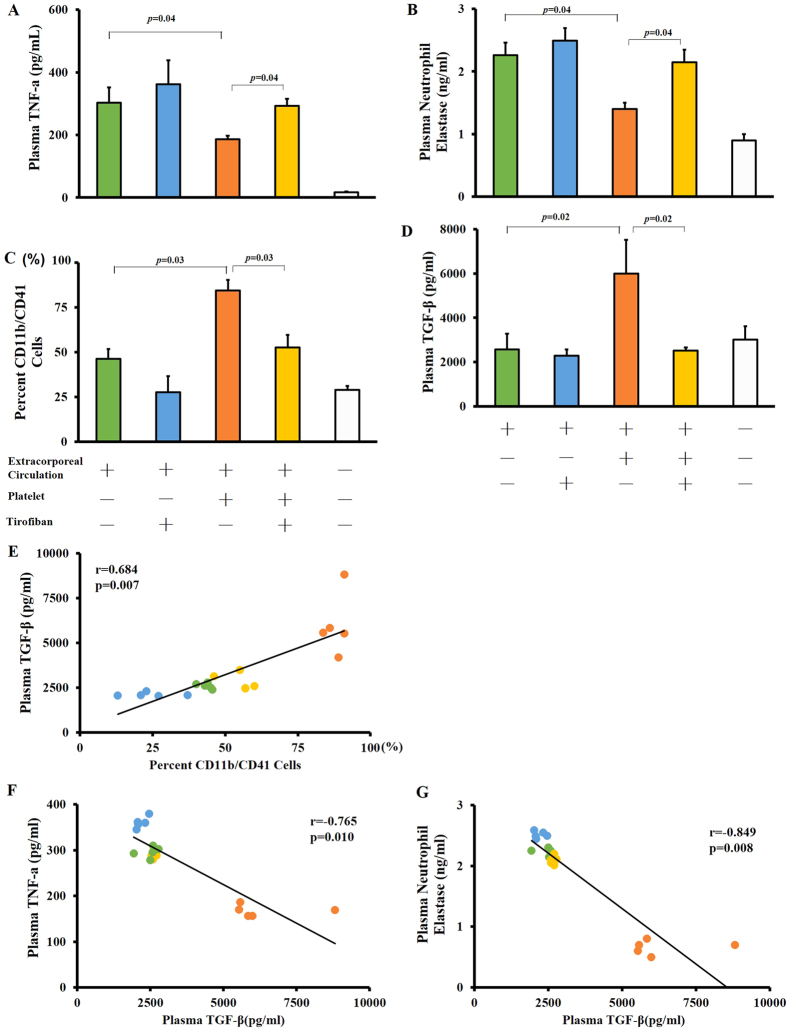
Platelet transfusion attenuates ECC-induced systemic inflammation. Animals were treated as described in the legend to Fig. 2, and their plasma was assayed for TNF-α (**A**), TGF-β (**B**), and neutrophil elastase (**D**) using commercial ELISA kits. The activation status of leukocytes was determined as up-regulation of CD11b. For platelet studies, the CD41-positive cell population was analyzed. The percentage of CD11b/CD41 cells, which provides an index of platelet-leukocyte aggregates, was determined by flow cytometry (**C**). Platelet transfusion led to significantly higher TGF-β levels, which were reduced by Tirofiban (**D**). Levels of TGF-β correlated positively with numbers of PLAs (**E**) and negatively with levels of both TNF-α (**F**) and NE (**G**). Data shown are mean ± SEM.

Since platelet transfusion was observed to block leukocyte filtration, we examined whether platelets inhibit leukocyte activation by TNF-α or NE, which is a key step in their migration into lung tissue^19^. ECC increased plasma levels of both TNF-α (p = 0.03, Fig. 5A) and NE (p = 0.03, Fig. 5B), which platelet transfusion significantly lowered. Tirofiban partly reversed these platelet effects. These results suggest that circulating platelets may help attenuate systemic inflammation.

Since activated platelets can adhere to leukocytes to form PLAs, we examined whether platelet transfusion increased the numbers of PLAs. Indeed, transfusing platelets significantly increased levels of PLAs (p = 0.03) in parallel with levels of TGF-β (p = 0.02), and these effects were reversed by Tirofiban (Fig. 5C).

Since platelets can release anti-inflammatory TGF-β when they interact with activated leukocytes, we examined whether the ability of platelet transfusion to increase the numbers of PLAs correlated with increased production of TGF-β. Indeed, platelet transfusion significantly increased TGF-β levels, and this effect was blocked by Tirofiban (p = 0.03, Fig. 5D). In addition, levels of TGF-β correlated positively with numbers of PLAs (p = 0.007, Fig. 5E), suggesting that transfused platelets were the major source of TGF-β. At the same time, levels of TGF-β correlated negatively with plasma levels of both TNF-α (p = 0.010, Fig. 5F) and NE (p = 0.008, Fig. 5G). These results suggest that circulating platelets exert anti-inflammatory effects, which are mediated at least partly by release of TGF-β from transfused platelets.

## Discussion

**Figure 6:**
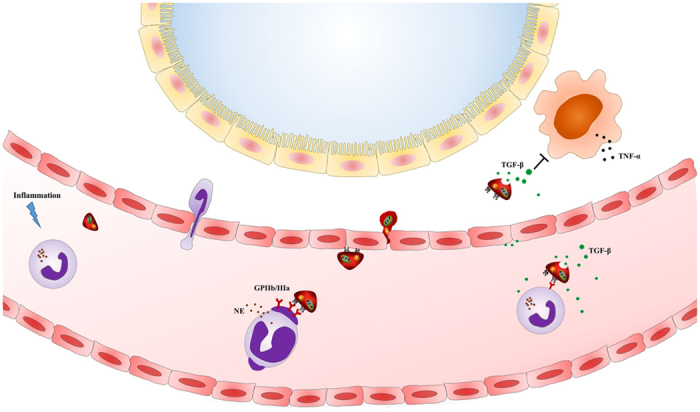
A model of how platelets alleviate ECC-induced inflammation. ECC induces a systemic inflammatory response, which activates platelets and neutrophils, leading the latter to degranulate and release pro-inflammatory factors into the lungs and blood. Following ECC, platelets migrate into lung tissue, where they release TGF-β and thereby inhibit pulmonary inflammation. At the same time, platelets release anti-inflammatory TGF-β into the blood.

The present study provides *in vivo* evidence that platelets transfused after ECC protect lung function by interacting with circulating activated leukocytes and migrating to lung tissue; the transfused platelets release TGF-β into the blood and within lung tissue, inhibiting systemic and pulmonary inflammatory responses (Fig. 6). These results raise the possibility that platelet transfusion may be a useful way to prevent and mitigate acute pulmonary dysfunction induced by systemic inflammatory responses.

Our results, combined with those of previous studies^7^, highlight the ability of platelets to bidirectionally regulate the inflammatory response. On one hand, platelet activation can promote inflammatory responses. Changes in temperature, contact with non-physiological surfaces such as tubing and systemic heparinization can activate circulating platelets during ECC^13^. This activation leads to the release of molecules from intracellular granules into the circulation as well as to the translocation of molecules to the platelet surface, where they mediate aggregation with other platelets or leukocytes. In clinical experiments^20^,^21^ and in our animal model, ECC-induced systemic inflammatory responses are associated with higher numbers of PLAs and higher levels of TNF-α and NE.

On the other hand, platelets can promote anti-inflammatory responses. We found that platelet transfusion strongly enhanced levels of TGF-β in circulation, and that these levels correlated positively with numbers of PLAs. At the same time, transfusion led to lower levels of both TNF-α and NE. Our findings suggest that TGF-β from transfused platelets helps counteract an excessive proinflammatory response following ECC.

Indeed, our experiments suggest that this anti-inflammatory mechanism directly mitigates acute pulmonary dysfunction induced by systemic inflammatory responses. Lung inflammation is characterized by the up-regulation of cytokine release from macrophages and monocytes residing in the lung interstitium, alveoli and airways. Key among these cytokines is TNF-α, which is secreted by activated macrophages, monocytes and neutrophils early in systemic inflammatory responses^22^. TNF-α acts as an initial inflammatory cytokine that subsequently regulates early neutrophil infiltration and helps recruit neutrophils from blood to sites of inflammation in the lung mesenchyma, as we observed in animals subjected to ECC. When we transfused the animals with platelets after ECC, the platelets localized to the lung interstitium, where levels of TGF-β were higher and levels of TNF-α and NE were lower than in control animals.

To assess whether interaction between platelets and endothelial cells is essential for transfused platelets to be able to migrate to lung tissue and attenuate inflammation there, we performed transfusion experiments in the absence or presence of the GP IIb/IIIa receptor antagonist Tirofiban. This inhibitor reduced the accumulation of platelets from GFP+ mice in lung interstitial tissue and led to more severe lung injury, suggesting that platelets must interact with endothelial cells in order to protect against injury resulting from systemic inflammatory responses.

This interaction may be related to mechanisms of leukocyte-platelet cross-talk via different receptor-ligands, which are only beginning to be understood. For example, platelet binding to the active region at the leading edge of polarized neurophils via interaction with Mac-1 (CD11b/CD18) regulates inflammatory pathways in one way, while platelet binding to the uropod via PSGL-1 regulates pathways in another way^7^. We found that inhibiting the interaction between GP IIb/IIIa on platelets and Mac-1 on leukocytes reduced the numbers of PLAs and enhanced both lung and systemic inflammatory responses, implying that the GPIIb/IIIa/Mac-1 signalling pathway helps mediate the anti-inflammatory effects of platelets.

Our results indicate a primarily anti-inflammatory role for the transfused platelets in our mouse model, begging the question of why the platelets show primarily those effects when numerous studies show them capable of *promoting* inflammation as well. For example, why were such different functional outcomes observed between the control and transfusion groups when they showed similar numbers of circulating platelets at 60 min after ECC? Further work is needed to clarify under what conditions platelets exert pro- or anti-inflammatory effects. The direction of effects may depend on mediators such as fibrinogen, which may act as a bridge to connect GP IIb/IIIa with Mac-1 following platelet activation^23^. The direction of effects may also depend on the timing of platelet transfusion. ECC increases levels of thrombin^24^, which is a potent activator of platelets. It is possible that transfusing platelets before and during ECC would not mitigate systemic inflammation-induced injury, or might even exacerbate it, given that exposing blood to nonphysiological surfaces such as ECC tubing increases levels of thrombin and of pro-inflammatory factors such as TNF-α^24^. This implies that administering platelets after ECC may help ensure that they exert anti-inflammatory effects since the platelets will not be activated by ECC-triggered increases in thrombin or TNF-α. Future studies should directly examine the effects of transfusing platelets before or simultaneously with ECC.

The source of TGF-β in our experiments is most likely the transfused platelets, since these cells have been shown to contain high TGF-β concentrations^9^ and to regulate TGF-β levels in circulation through secretion or degranulation^25^. Though leukocytes cultured *in vitro* also secrete cytokines, there is no direct evidence that such cells secrete TGF-β into plasma^26^. Nevertheless, we cannot entirely exclude the possibility that the observed increase in TGF-β comes from endogenous sources, such as leukocytes, endothelial cells, or even endogenous circulating platelets, perhaps in response to stimulation by the transfused platelets. Further work should directly examine the sources of TGF-β production following platelet transfusion.

The present study suggests that platelets play a critical role in mitigating systemic inflammatory responses and consequent acute pulmonary dysfunction after ECC. Future studies should verify and extend our findings by analyzing outcomes at times shorter and longer than 60 min in order to understand the processes behind platelet-mediated attenuation of lung injury. For example, it is unclear why numbers of circulating platelets in the control group fell below baseline within 5 min after ECC, and then later recovered to similarly high levels as in the transfusion group. It is possible that platelets are eventually released from organs such as the spleen into circulation. Future work should also examine the effects of transfusing different amounts of platelets: using too few may fail to give clinically significant effects, while using too many may raise safety concerns. Finally, future studies should examine how the dose and timing of platelet transfusion—as well as other factors—must be controlled in order to shift platelet-mediated bidirectional regulation of inflammatory responses towards net anti-inflammatory effects. Such work may lead to novel approaches to prevent and manage lung injury induced by systemic inflammatory responses.

## Methods and Materials

### Animal model

This work involved our previously described mouse model of lung injury induced by a systemic inflammatory response following ECC^13^. Briefly, C57BL/6 mice were anesthetized by intraperitoneal administration of 50 mg/kg pentobarbital, intubated orotracheally with a 20-gauge angiocatheter (Becton Dickinson Medical Devices, USA), and then connected to a small rodent ventilator (Taimeng Keji, Chengdu, China). The respiratory rate was set at 130 breaths/min, and the tidal volume was 0.5 ml of room air. Surgical procedures were performed with the aid of an operating microscope offering 10–40x magnification (Precision Stereo Zoom Trinocular Microscope III, World Precision Instruments, USA). The right carotid artery was dissected out between the right strap muscle and sternocleidomastoid muscle. The omohyoid muscle was cut to improve visibility during the surgical procedure. After administration of heparin (500 U/kg), a 24-gauge intravenous catheter (Becton Dickinson Medical Devices) was inserted into the right carotid artery and another was inserted into the external jugular vein. To set up the ECC, these two catheters were connected with a tube (inner diameter, 1/32 inch) primed with 0.4 mL normal saline and attached to a roller pump (Stock II, Munich, Germany). The flow rate was maintained at 5 ml/min, corresponding to approximately 25% of cardiac output. Our previous work^13^ has shown that this procedure does not alter the hemodynamics of the mice. All animal experimental protocols were performed in accordance with the Guide for the Care and Use of Laboratory Animals prepared by the Institutional Animal Care and Use Committee of Sichuan University. Experimental protocols were approved by Sichuan University.

### Study design

In experiments to examine the effects of ECC on platelet count, mice underwent ECC for 30 min and then immediately received phosphate-buffered saline (PBS, 0.1 ml) or platelets (0.1 ml, 3 × 10^8^ from 1.5 ml blood). They were sacrificed at 60 min later (n = 20 in each group). Additional animals (n = 20) were sacrificed at 5 min to assay the number of transfused platelets. To provide baseline data, 20 animals were anesthetized and sacrificed without ECC.

In experiments to examine the effects of platelets on the systemic inflammatory response and associated lung injury, mice underwent ECC for 30 min, after which they were treated with platelets (0.1 ml, 3 × 10^8^ from 1.5 ml blood), Tirofiban in PBS (0.1 ml, 60 μg/kg; Sigma-Aldrich, St. Louis, USA), the combination of platelets and Tirofiban, or PBS (n = 10 per group). After 60 min, blood and lung tissues were harvested and assessed for inflammatory cytokine levels and organ function. Control mice (n = 10) were naïve to any intervention and were analyzed in the same way.

### Lung function and histological injury

At the end of the post-ECC observation period, arterial blood samples were taken from the carotid artery, and O_2_ tension (PaO_2_) was measured. Lung function was assessed by measuring FiO_2_/PaO_2_ ratio; FiO_2_ was assumed to be 0.21. Lung injury was assessed histopathologically by harvesting the left lung and fixing overnight in 4% paraformaldehyde at 4 °C. Paraffin-embedded sections were stained with hematoxylin and eosin and examined under a light microscope by a pathologist blinded to the experimental groups. Severity of lung injury was scored using a 5-point scale (0 = normal histology, 5 = most severe injury) that took into account alveolar congestion, haemorrhage, neutrophil accumulation in the airspace or vessel wall, alveolar wall thickness, and hyaline membrane formation. The mean score per section was calculated for each animal.

### Platelet preparation

Blood (1.5 ml) was collected from donor mice immediately before ECC and mixed with 0.1 ml of acid-citrate-dextrose buffer (Sigma-Aldrich, USA). Platelets were isolated as described previously^27^. Briefly, blood was centrifuged at 100 *g* for 10 min to collect the supernatant of platelet-rich plasma (PRP). Platelets were then pelleted from PRP by centrifugation at 740 *g* for 10 min. The platelet pellets were gently resuspended in 500 μl of PBS. For cell counting, platelets were loaded onto a haemocytometer and left to settle for 30 minutes. The final concentration was adjusted to 3 × 10^8^ in 0.1 ml for transfusion during experiments. Based on flow cytometry of cells labelled with anti-CD62 and anti-CD41 antibodies, <1% of isolated platelets were activated at the time of transfusion.

### Inflammatory cytokine levels in plasma and lung

To measure plasma markers of ECC-induced systemic inflammation in our model, blood samples were obtained at the end of the post-ECC observation period. Plasma was isolated by centrifuging the total sample for 15 min at 1000 *g* at 4 °C, and the plasma supernatant was removed and stored at −70 °C until analysis. Commercial ELISA kits were used to assay plasma levels of TNF-α and TGF-β (Thermo Scientific, Rockford, IL, USA) as well as neutrophil elastase (NE; Cloud-Clone, Houston, TX, USA).

To measure ECC-induced inflammatory changes of lung tissue in our model, the right lung was harvested, homogenized, and centrifuged as described^13^. Briefly, tissues were harvested on ice immediately after euthanasia and weighed. An aliquot of tissue (50 mg) was cut into 1-mm^3^ pieces, added to 500 μl PBS and homogenized. Samples were left standing on ice for 5 min and centrifuged at 3500 *g* for 20 min at 4 °C. Supernatants were transferred to Eppendorf tubes and stored at −70 °C until analysis, when they were assayed for TNF-α, TGF-β, and NE as described above for plasma samples.

### Flow cytometric analysis of PLAs

Blood samples were centrifuged at 100 *g* for 10 min, and the supernatant of PRP was discarded. The pelleted erythrocytes were lysed with ammonium chloride lysis solution at room temperature for 10 min, then washed 3 times in PBS. The activation status of leukocytes was determined as increased binding of PE-conjugated anti-CD11b antibody (eBioscience, San Diego, USA). For platelet studies, the population of cells positive for FITC-conjugated anti-CD41 antibody (eBioscience, San Diego, USA) was analyzed. Cells (1 × 10^6^) were incubated at 4 °C for 1 h with anti-CD11b and anti-CD41 antibodies, washed twice in 1 ml PBS, re-suspended in 500 μl PBS, and then analyzed by flow cytometry on an Esp Elite device (Beckman Coulter, Chicago, IL, USA). IgG1-FITC/PE antibody (eBioscience, San Diego, USA) served as a negative control.

### Confocal microscopy

To track the migration of transfused platelets, platelets were harvested from GFP-labelled mice (n = 4) as described above and injected after ECC. At the end of the observation period, the left lung was harvested and fixed overnight at 4 °C in 4% paraformaldehyde. Paraffin-embedded sections were analysed by confocal microscopy. The number of GFP-labelled platelets in lung tissue was counted in 200 fold microscopic fields.

### Statistical analysish

Statistical analysis was performed using SPSS 11.01 (IBM, Chicago, USA). Results were reported as mean ± SEM. Differences between two groups were assessed for significance using Student’s *t* test; differences between more than two groups were assessed using one-way ANOVA. Pearson correlation analysis was used to identify associations between variables. The threshold for significance in all statistical tests was p < 0.05.

## Additional Information

**How to cite this article**: Luo, S. *et al*. Platelets protect lung from injury induced by systemic inflammatory response. *Sci. Rep.* **7**, 42080; doi: 10.1038/srep42080 (2017).

**Publisher's note:** Springer Nature remains neutral with regard to jurisdictional claims in published maps and institutional affiliations.

## References

[b1] Smail N. . Role of systemic inflammatory response syndrome and infection in the occurrence of early multiple organ dysfunction syndrome following severe trauma. Intensive care medicine 21, 813–816 (1995). 8557869 10.1007/BF01700964

[b2] Asimakopoulos G., Smith P. L., Ratnatunga C. P. & Taylor K. M. Lung injury and acute respiratory distress syndrome after cardiopulmonary bypass. The Annals of thoracic surgery 68, 1107–1115 (1999). 10510030 10.1016/s0003-4975(99)00781-x

[b3] Diacovo T. G., Roth S. J., Buccola J. M., Bainton D. F. & Springer T. A. Neutrophil rolling, arrest, and transmigration across activated, surface-adherent platelets via sequential action of P-selectin and the beta 2-integrin CD11b/CD18. Blood 88, 146–157 (1996). 8704169

[b4] Weber C. & Springer T. A. Neutrophil accumulation on activated, surface-adherent platelets in flow is mediated by interaction of Mac-1 with fibrinogen bound to alphaIIbbeta3 and stimulated by platelet-activating factor. The Journal of clinical investigation 100, 2085–2093, doi: 10.1172/JCI119742 (1997). 9329974 PMC508400

[b5] Evangelista V. . Platelet/polymorphonuclear leukocyte interaction in dynamic conditions: evidence of adhesion cascade and cross talk between P-selectin and the beta 2 integrin CD11b/CD18. Blood 88, 4183–4194 (1996). 8943853

[b6] Kaplan Z. S. . Thrombin-dependent intravascular leukocyte trafficking regulated by fibrin and the platelet receptors GPIb and PAR4. Nature communications 6, 7835, doi: 10.1038/ncomms8835 (2015). 26204458

[b7] Sreeramkumar V. . Neutrophils scan for activated platelets to initiate inflammation. Science 346, 1234–1238, doi: 10.1126/science.1256478 (2014). 25477463 PMC4280847

[b8] Valerio-Rojas J. C., Jaffer I. J., Kor D. J., Gajic O. & Cartin-Ceba R. Outcomes of Severe Sepsis and Septic Shock Patients on Chronic Antiplatelet Treatment: A Historical Cohort Study. Critical Care Research and Practice 2013, 9, doi: 10.1155/2013/782573 (2013). PMC3590611 23509620

[b9] Peracoli M. T. . Platelet aggregation and TGF-beta(1) plasma levels in pregnant women with preeclampsia. Journal of reproductive immunology 79, 79–84, doi: 10.1016/j.jri.2008.08.001 (2008). 18805591

[b10] Gudbrandsdottir S., Hasselbalch H. C. & Nielsen C. H. Activated platelets enhance IL-10 secretion and reduce TNF-alpha secretion by monocytes. Journal of immunology 191, 4059–4067, doi: 10.4049/jimmunol.1201103 (2013). 24048901

[b11] Xiang B. . Platelets protect from septic shock by inhibiting macrophage-dependent inflammation via the cyclooxygenase 1 signalling pathway. Nature communications 4, 2657, doi: 10.1038/ncomms3657 (2013). PMC4217311 24150174

[b12] Van Poucke S. . Early platelet recovery following cardiac surgery with cardiopulmonary bypass. Platelets, 1–7, doi: 10.3109/09537104.2016.1173665 (2016). 27164510

[b13] Luo S. . A novel minimal invasive mouse model of extracorporeal circulation. Mediators of inflammation 2015, 412319, doi: 10.1155/2015/412319 (2015). 25705092 PMC4325217

[b14] Du L. . Actin filament reorganization is a key step in lung inflammation induced by systemic inflammatory response syndrome. American journal of respiratory cell and molecular biology 47, 597–603, doi: 10.1165/rcmb.2012-0094OC (2012). 22721831

[b15] Bombeli T., Schwartz B. R. & Harlan J. M. Adhesion of activated platelets to endothelial cells: evidence for a GPIIbIIIa-dependent bridging mechanism and novel roles for endothelial intercellular adhesion molecule 1 (ICAM-1), alphavbeta3 integrin, and GPIbalpha. The Journal of experimental medicine 187, 329–339 (1998). 9449713 10.1084/jem.187.3.329 PMC2212123

[b16] Lesurtel M. . Platelet-derived serotonin mediates liver regeneration. Science 312, 104–107, doi: 10.1126/science.1123842 (2006). 16601191

[b17] Massoudy P. . Evidence for inflammatory responses of the lungs during coronary artery bypass grafting with cardiopulmonary bypass. Chest 119, 31–36 (2001). 11157581 10.1378/chest.119.1.31

[b18] Li M. O., Wan Y. Y., Sanjabi S., Robertson A. K. & Flavell R. A. Transforming growth factor-beta regulation of immune responses. Annual review of immunology 24, 99–146, doi: 10.1146/annurev.immunol.24.021605.090737 (2006). 16551245

[b19] Burch R. M., Noronha-Blob L., Bator J. M., Lowe V. C. & Sullivan J. P. Mice treated with a leumedin or antibody to Mac-1 to inhibit leukocyte sequestration survive endotoxin challenge. Journal of immunology 150, 3397–3403 (1993). 8468478

[b20] Guay J., Ruest P. & Lortie L. Cardiopulmonary bypass induces significant platelet activation in children undergoing open-heart surgery. European journal of anaesthesiology 21, 953–956 (2004). 15719858 10.1017/s026502150400033x

[b21] Celik J. B., Gormus N., Okesli S., Gormus Z. I. & Solak H. Methylprednisolone prevents inflammatory reaction occurring during cardiopulmonary bypass: effects on TNF-alpha, IL-6, IL-8, IL-10. Perfusion 19, 185–191 (2004). 15298427 10.1191/0267659104pf733oa

[b22] Schulte W., Bernhagen J. & Bucala R. Cytokines in sepsis: potent immunoregulators and potential therapeutic targets–an updated view. Mediators of inflammation 2013, 165974, doi: 10.1155/2013/165974 (2013). 23853427 PMC3703895

[b23] Patko Z., Csaszar A., Acsady G., Peter K. & Schwarz M. Roles of Mac-1 and glycoprotein IIb/IIIa integrins in leukocyte-platelet aggregate formation: stabilization by Mac-1 and inhibition by GpIIb/IIIa blockers. Platelets 23, 368–375, doi: 10.3109/09537104.2011.625098 (2012). 22671289

[b24] Ernofsson M., Thelin S. & Siegbahn A. Monocyte tissue factor expression, cell activation, and thrombin formation during cardiopulmonary bypass: a clinical study. The Journal of thoracic and cardiovascular surgery 113, 576–584, doi: 10.1016/S0022-5223(97)70373-8 (1997). 9081105

[b25] Grainger D. J., Mosedale D. E. & Metcalfe J. C. TGF-beta in blood: a complex problem. Cytokine & growth factor reviews 11, 133–145 (2000). 10708961 10.1016/s1359-6101(99)00037-4

[b26] Assoian R. K. . Expression and secretion of type beta transforming growth factor by activated human macrophages. Proceedings of the National Academy of Sciences of the United States of America 84, 6020–6024 (1987). 2888109 10.1073/pnas.84.17.6020 PMC298999

[b27] Li Z., Rumbaut R. E., Burns A. R. & Smith C. W. Platelet response to corneal abrasion is necessary for acute inflammation and efficient re-epithelialization. Investigative ophthalmology & visual science 47, 4794–4802, doi: 10.1167/iovs.06-0381 (2006). 17065490

